# Hypoxia signalling in the regulation of innate immune training

**DOI:** 10.1042/BST20210857

**Published:** 2021-01-11

**Authors:** Lauren Eades, Michael Drozd, Richard M. Cubbon

**Affiliations:** Leeds Institute of Cardiovascular and Metabolic Medicine, The University of Leeds, Clarendon Way, Leeds LS2 9JT, U.K.

## Abstract

Innate immune function is shaped by prior exposures in a phenomenon often referred to as ‘memory’ or ‘training’. Diverse stimuli, ranging from pathogen-associated molecules to atherogenic lipoproteins, induce long-lasting training, impacting on future responses, even to distinct stimuli. It is now recognised that epigenetic modifications in innate immune cells, and their progenitors, underpin these sustained behavioural changes, and that rewired cellular metabolism plays a key role in facilitating such epigenetic marks. Oxygen is central to cellular metabolism, and cells exposed to hypoxia undergo profound metabolic rewiring. A central effector of these responses are the hypoxia inducible factors (or HIFs), which drive transcriptional programmes aiming to adapt cellular homeostasis, such as by increasing glycolysis. These metabolic shifts indirectly promote post-translational modification of the DNA-binding histone proteins, and also of DNA itself, which are retained even after cellular oxygen tension and metabolism normalise, chronically altering DNA accessibility and utilisation. Notably, the activity of HIFs can be induced in some normoxic circumstances, indicating their broad importance to cell biology, irrespective of oxygen tension. Some HIFs are implicated in innate immune training and hypoxia is present in many disease states, yet many questions remain about the association between hypoxia and training, both in health and disease. Moreover, it is now appreciated that cellular responses to hypoxia are mediated by non-HIF pathways, suggesting that other mechanisms of training may be possible. This review sets out to define what is already known about the topic, address gaps in our knowledge, and provide recommendations for future research.

## Introduction

Whilst the adaptive immune system was originally defined by its capacity to retain a specific memory of prior exposure to pathogens, accumulating evidence suggests that the innate immune system also possesses a primitive non-selective form of memory. In 2011, Mihai Netea et al. [1] summarised the published literature supporting this concept and coined the term ‘trained immunity’, stimulating a large body of subsequent research showing the broad relevance of this phenomenon to innate immune function. Notably, trained immunity can be induced by diverse stimuli (e.g. β-glucan, oxidised LDL-cholesterol, catecholamines), is retained over periods longer than the lifespan of most mature innate immune cells, and alters the innate immune response to multiple distinct secondary stimuli [2–4]. The search for how diverse training stimuli could induce sustained non-specific adaptations soon turned to epigenetics, and it became clear that the metabolic responses to primary stimuli could indeed lead to training via epigenetic modifications [4], although it is important to note that other factors, such as cAMP signalling, also mediate some epigenetic responses [5]. Whilst there is not a unique metabolic profile underpinning training-induced epigenetic modifications [6–9], a commonly implicated contributor is augmented aerobic glycolysis, often associated with wider changes such as altered tricarboxylic acid cycle flux and/or glutaminolysis. For example, detection of β-glucan (a fungal cell wall component) by monocyte pattern recognition receptors initiates a signalling cascade via Akt and mTOR to HIF-1α (hypoxia inducible factor-1α), promoting aerobic glycolysis [4]. HIF-1α orchestrates wide-ranging changes in the transcription of metabolic genes and was already known to be essential for myeloid cell function and pathogen responses [10–12]. As detailed later, the seminal work of Cheng et al. [4] added facilitation of trained immunity to this repertoire. However, it is important to emphasise that other potent training stimuli, such as Bacillus Calmette-Guerin (BCG) and bacterial lipopolysaccharide (LPS), induce somewhat distinct metabolic responses to β-glucan [13,14], so it cannot be assumed that HIF-1α participates in these circumstances. Indeed, many uncertainties remain about the role of hypoxia signalling and hypoxia in trained immunity and this review aims to summarise existing literature, along with important unresolved or unexplored questions, with a particular focus on monocyte and macrophage biology.

## Hypoxia signalling in myeloid cells

The importance of cellular oxygen sensing and adaptation is emphasised by the 2019 award of the Nobel Prize in Physiology or Medicine [15]. In part, this award recognised work showing that HIF proteins are targeted for proteasomal degradation under normoxic circumstances due to hydroxylation by one of the Prolyl Hydroxylase Domain (PHD) family of enzymes. This prevents HIFs from acting in their role as transcription factors that regulate gene expression programmes required for adaptation to hypoxia [15]. There are three HIF-α isoforms, all of which are expressed in myeloid cells; little is known about the role of HIF-3α in this lineage, but it is generally thought to act as a negative regulator of HIF-1α and HIF-2α signalling [16,17]. Importantly, HIF-1α and HIF-2α have overlapping but distinct roles in myeloid transcriptional regulation, allowing a broader range of context-specific hypoxia responses [18]. Myeloid HIF-1α knockout mice have a complex phenotype, with altered macrophage function, including reduced: motility; phagocytosis of bacteria; and participation in chronic inflammation [12,19]. Whilst HIF-1α knockout macrophages have impaired cytokine release in response to group A streptococci, their response to tetradecanoyl phorbol acetate is unaltered, emphasising the context-dependent role of HIF-1α. These phenotypes are associated with impaired promotion of myeloid glycolysis and a substantial reduction in adenosine triphosphate generation [12,19]. Myeloid HIF-2α knockout mice also have impaired LPS responses [20]. The phenomenon of macrophage skewing, whereby stereotyped phenotypic characteristics are induced by specific stimuli (e.g. LPS or IFNγ for M1 phenotype [classically activated] and IL4 or IL13 for M2 phenotype [alternatively activated]) is also linked to HIF biology. Indeed, Takeda et al. demonstrated that M1 skewing was associated with HIF-1α induction, whereas M2 was associated with greater HIF-2α induction, with alterations in these isoforms being responsible for changes in the expression of iNOS and Arginase-1, key markers of M1 and M2 phenotype, respectively [21]. Whilst the M1/M2 dichotomy oversimplifies macrophage biology, describing rather artificial *in vitro* phenotypes that do not reflect *in vivo* complexity, these data illustrate that diverse phenotypes can arise from hypoxia signalling, depending on the context of HIF-α isoform abundance.

Given the crucial role of oxygen in cell biology, it is unsurprising that factors beyond PHD-mediated regulation of HIF degradation contribute to hypoxia sensing. Firstly, it is important to note that PHDs may hydroxylate non-HIF targets, affecting their degradation or activity in response to oxygen availability [22]; this broadens the repertoire of PHDs in matching cellular responses to oxygenation. One pertinent example of this is PHD1-mediated hydroxylation of IKKβ, which promotes the ability of IKKβ to inhibit the transcriptional activity of NFkB, thereby suppressing NFkB-induced pro-inflammatory gene programmes [23]. It is also known that HIFs can be hydroxylated (at different residues to those targeted by PHDs) by Factor Inhibiting HIF (FIH), another oxygen sensing enzyme that relies on 2-oxoglutarate as a cofactor, but is structurally distinct to PHDs [24]. FIH-mediated asparagine hydroxylation of HIFs instead inhibits their binding to the transcriptional co-activator complex, CREB-binding protein (CBP)/p300, hindering induction of gene expression [25]. FIH is known to modulate HIF activity in myeloid cells [26], and, like PHDs, also hydroxylates other non-HIF proteins [27], although the functional consequences of the latter is unclear. Another more recently emerging family of oxygen sensing enzymes are the Jumonji C domain histone lysine demethylases (JmjC-KDMs); as their name implies, these demethylate the chromatin regulating histone proteins, thereby altering DNA accessibility and gene expression [28,29]. Many family members are expressed in myeloid cells, and although the precise role of most is unexplored, some have important roles in immune training, as discussed later.

## Hypoxic versus normoxic hypoxia signalling in myeloid cells

HIF-1α is well established to modulate cellular glucose metabolism by promoting the transcription of membrane glucose transporters and most glycolytic enzymes. It also suppresses downstream glucose oxidation, for example by augmenting expression of pyruvate dehydrogenase kinase-1 (which hinders pyruvate dehydrogenase's catalysis of pyruvate to acetyl-CoA) and suppressing expression of mitochondrial cytochrome c subunit 4. Hence, a major metabolic impact of HIF-1α activation is increased glycolysis, as described in a number of excellent reviews [30,31], although many other metabolic pathways are also influenced [32]. It is also important to note that whilst HIF-activating stimuli often diminish oxidative phosphorylation and increase glycolysis [4], some stimuli may increase both oxidative phosphorylation and glycolysis, emphasising the importance of context [33]. Crucially, HIF-1α signalling can be induced under normoxic conditions - indeed, the majority of the literature linking HIF signalling to immune cell function is underpinned by experimental data collected in normoxic conditions. Stimulation of macrophages with LPS in normoxia increases the expression [34], reduces the degradation [10], and promotes the transcription factor activity of HIF-1α [35]. Many complex mechanisms underpin these observations, although it is particularly interesting to note that LPS-induced accumulation of succinate, a Krebs cycle intermediate, results in succinate-mediated inhibition of PHD enzymes, thereby retarding HIF-1α degradation during normoxia [10]. This is a useful illustration of how cellular metabolic status modulates HIF signalling, rather than just being passively influenced by HIFs, emphasising their complex bidirectional relationship. Notably, this normoxic rewiring of glucose metabolism is essential for the inflammatory cytokine response that occurs in response to stimuli like LPS [10]. Interestingly, HIF-2α activity appears not to promote glycolysis, which may underpin the previously discussed distinct roles of HIF-1α and HIF-2α in macrophage biology [20,31].

A commonly observed theme in normoxic HIF activation is the involvement of increased signalling via Akt and mTOR [4], both major regulators of metabolism [36], with important influences on myeloid behaviour and HIF-1α signalling [37]. Other pro-inflammatory signalling nodes are also likely to play an important role in normoxic HIF induction, with NFkB and p42/44 mitogen-activated protein kinase (ERK) being examples of signalling nodes able to promote HIF-1α signalling [38–40]. Particularly important to mention here is the family of pro-inflammatory interleukin-1 (IL-1) cytokines, which are essential for innate immune responses and can signal via Akt, mTOR, ERK, and NFkB, and lead to increased HIF-1α activity [41]. Unsurprisingly, IL-1 signalling augments glycolysis in many cell lineages [42], and has wide-ranging HIF-dependent effects on myeloid cell function, as will be discussed later [41]. Conversely, glycolysis (a HIF-1α induced process) is required to promote the activation of the IL-1β isoform [43], further highlighting the multi-directional cross-talk between hypoxia signalling, metabolism and inflammation.

Whilst there is abundant literature describing the influence of hypoxia on myeloid cells, much of this was collected before the details of HIF signalling were uncovered. For example, nearly 40 years ago Knighton et al. [44] described how macrophage culture in hypoxia induces secretion of pro-angiogenic factors. Subsequent studies have described much broader changes in macrophages cultured in hypoxia, such as altered: cytokine secretion [45], nitric oxide generation [46], glycolysis [47], redox status [48], migration [49], and polarisation [50]. Hence, it is clear that hypoxia has wide-ranging effects on myeloid cells, but the role of hypoxia sensing and signalling mechanisms in these responses is poorly understood, leaving a large gap in our understanding. Perhaps more important, we do not understand how hypoxic and normoxic activation of hypoxia signalling differ from one another, limiting our ability to translate these findings to clinical benefit. However, a recently published macrophage proteomic dataset provides some interesting insights [50]. This revealed that the signatures induced by 3% hypoxia versus normoxic LPS/IFNγ stimulation are by no means identical, although the induction of the glycolytic enzyme hexokinase-2 (a HIF-1α regulated gene) was similar in the two conditions. Further analyses of such datasets could provide valuable insights by focussing on known HIF targets.

Beyond our limited awareness of hypoxic HIF signalling, there are also major gaps in our understanding of how other oxygen sensing systems might modulate myeloid cell function (either in hypoxia or normoxia), although it is possible to speculate. For example, DNA methylation, a form of epigenetic modification, is influenced by the oxygen sensing family of ten-eleven translocation (TET) 2-oxoglutarate-dependent enzymes, which catalyse DNA demethylation [51]. Notably, mutations in TET2 are commonly implicated in clonal haematopoiesis of indeterminate potential, a common premalignant clonal disorder of the myeloid compartment [52], associated with a pro-inflammatory state that promotes cardiovascular disease [52,53]. Regulation of RNA demethylation is also governed by a separate family of oxygen sensing enzymes; notably, mutations in one of these, FTO Alpha-Ketoglutarate Dependent Dioxygenase, are again implicated in myeloid malignancy [54]. Whilst other oxygen sensing enzymes exist, such as cysteamine 2-aminoethanethiol dioxygenase, the role of these in myeloid cells is unknown.

It is evident that cellular oxygen sensing apparatus are linked with cellular metabolism at multiple levels. Moreover, hypoxia is generally accompanied by wide-ranging changes in cellular energetic status and metabolic substrate availability/preference. Hence, it is important to view hypoxia signalling in a broader context of metabolic perturbation, the context of which is likely to modify the nature and outcome of hypoxia sensing, for example as described in the context of normoxic versus hypoxic HIF signalling. Indeed, it is possible that normoxic HIF activation may even promote supra-normal oxygen availability [55]. Adding further complexity, hypoxia and associated metabolic disturbances are often encountered in disease contexts where factors like pathogens and/or ischaemic tissue necrosis add to the complexity of the environment and the resultant response to hypoxia. The relevance to disease states will be discussed later, but first we will review what is known about how hypoxia signalling influences, and is influenced by, innate immune training.

## Hypoxia signalling in immune training

It is important to emphasise that much of the published literature linking hypoxia signalling to immune training relies on *in vitro* studies conducted in normoxia, or *in vivo* studies where it is difficult to define oxygen tensions experienced by innate immune cells. Hence, whilst hypoxia is likely to modulate immune training, this assertion is predominantly based on circumstantial data. The first illustration of canonical hypoxia sensing systems being required for innate immune training came from Cheng et al. [4] who found that myeloid HIF-1α knockout mice lacked β-glucan training and died more often after subsequent induction of *S. aureus* septicaemia. Their working mechanistic model was that Akt/mTOR signalling upon β-glucan training augmented HIF-1α expression and activity, along with rewiring cellular metabolism, particularly with increased glycolysis. These signalling events were linked to marked H3K4me3 and H3K27Ac epigenetic modifications involving innate immune genes, although the causal relationship between these modifications and signalling or metabolic events was not unpicked. Subsequent work from this group has shown that Akt/mTOR signalling, glycolysis (and glutaminolysis) are also required for BCG training of monocytes, although the role of HIF-1α was never formally assessed in this study [14]. Interestingly, the training responses induced by β-glucan and BCG both require IL-1 signalling [56–58], the inhibition of which interferes with the generation of the epigenetic profile linked to training [57]. Notably, genetic variants in the *IL1B* gene (encoding IL-1β) have been linked to altered human training responses adding further weight to its importance [57,59].

Beyond HIFs, JmjC-KDMs are another element of the hypoxia sensing apparatus that influence innate immune training. This was first demonstrated by Arts et al. [13] who found that monocyte KDM5 participates in the integration of metabolic signals during innate immune training to induce epigenetic memory. This work uncovered an essential role of glycolysis and glutaminolysis in monocytes undergoing normoxic β-glucan training, which promoted the accumulation of permissive histone marks (H3K4me3) at pro-inflammatory gene promoters [13]. This was associated with the accumulation of fumarate, which stabilises and actives HIF, promoting pro-inflammatory gene expression. However, fumarate also reproduced much of the β-glucan-induced H3k4me3 profile, which suggested an additional epigenetic mechanism, independent of HIFs; this was attributed to the inhibitory effect of fumarate upon KDM5 transcription and activity. Importantly this phenomenon appeared after β-glucan priming, but not after LPS priming, possibly explaining their training versus tolerogenic effects, respectively. Subsequent work has suggested that β-glucan induced miR-9-5p expression may mediate many of the early metabolic responses via suppression of isocitrate dehydrogenase 3α [60]. Later, Liu et al. [61] showed that KDM6B facilitates normoxic M2 skewing in response to glutaminolysis-derived 2-oxoglutarate, by removing repressive H3K27me3 histone modifications from M2 marker genes. Notably, when macrophages were trained with LPS in glutamine free conditions and then later re-challenged with LPS, they exhibited greater release of pro-inflammatory cytokines and a more M1 skewed phenotype then cells trained with LPS in the presence of glutamine. Hence, KDM6B appears to impart a tolerogenic immune training phenotype via its histone-modifying effects. Overall, these data suggest that members of the oxygen-sensitive JmjC-KDM family are important epigenetic regulators of innate immune training and tolerance during normoxia, although the role that they play in hypoxia is unexplored.

Bekkering et al. [62] have separately shown that mevalonate, generated in the proximal cholesterol biosynthetic pathway, is required for induction of monocyte training in response to oxLDL, BCG and β-glucan. Expression of insulin-like growth factor-1 receptor (notably a HIF-regulated gene) was essential for mevalonate-induced training, and was shown to promote mTOR activation and glucose metabolism required for this phenotype. Notably, mevalonate induced H3K27ac epigenetic modifications mimicking those of β-glucan, illustrating another metabolite (beyond fumarate, described above) capable of directly training monocytes. Finally, recent data show that lactate, generated by anaerobic metabolism or via M1 skewing in normoxia can directly induce epigenetic changes in macrophages via histone lactylation, although this study did not directly assess subsequent training responses [63]. It is likely that many more metabolic intermediates will prove to influence training, based on wider literature linking cellular metabolism with epigenetics [64]. Furthermore, the wider context of cellular metabolic state and signalling milieu are likely to be important modifiers of hypoxia signalling and responses, meaning that there is probably no single stereotyped hypoxia response.

Whilst this review has focussed on monocytes and macrophages (with other excellent reviews describing neutrophils) [65,66], it is also important to discuss myeloid progenitor cells (MPCs) and haematopoietic stem cells (HSCs), since training responses can outlive mature myeloid cells. It is well established that diverse stimuli (including BCG, LPS and Western diet) induce sustained epigenetic modifications in MPCs and HSCs [59,67,68], resulting in altered innate immunity of recipients receiving HSC transplantation from trained donors [67,68]. Whilst most of these studies did not uncover essential molecular pathways for MPC/HSC training, some recent data provide useful insights. First, Mitroulis et al. [69] explored how β-glucan induces training in MPC/HSC, given that these cells cannot directly sense this molecule. They found an important paracrine/endocrine role for IL-1β in conveying this signal to MPC/HSC, leading to increased glycolysis and functional adaptations; whilst the role of HIF signalling was not directly explored, this seems plausible based on the data described earlier. These data emphasise the broad relevance of IL-1 signalling in training responses across multiple cell lineages. Second, de Laval et al. [67] implicated the transcription factor C/EBPβ in LPS-induced HSC training. This is interesting from the perspective of hypoxia signalling, since HIF-1α is well known to interact with C/EBPβ [70], although further studies are required to test this possibility. However, it is notable that HSCs are adapted to their specialised hypoxic bone marrow niche [71], and so if hypoxia signalling is found to regulate training at the level of HSCs, it will be important to consider how what constitutes and mediates the normal versus the training response to hypoxia. Beyond myeloid cells and their stem/progenitor hierarchy, training is known to occur in other lineages, such as epithelial cells, and it will also be important to uncover whether hypoxia signalling modulates training in these. Finally, whilst hypoxia signalling appears intrinsically linked to many training responses, we remain unclear of how many of the aforementioned oxygen sensing systems (such as HIF-2α and TET2) contribute to training in any cell lineage. Hence, there are many important unresolved questions about how hypoxia signalling influences trained immunity and answering these will be important to achieve translational benefit.

## Clinical and translational relevance

Immune training and epigenetic modification of innate immune function are gaining increasing attention in the clinical arena [72], with recent data even showing that BCG-vaccination can reduce future all-cause infection events in the elderly [73]. Notably, many disease states involving innate immune responses are also linked with altered local or systemic oxygen tension, such as: myocardial infarction (MI), cancer, infection, chronic lung disease, and obesity, to name a few. Using animal models, it has also been shown that training induced by MI can improve sepsis outcomes [74], but promotes breast cancer and atherosclerosis progression [75,76], illustrating how training also links seemingly distinct pathological processes. Importantly, monocytes from patients with acute coronary syndromes (which includes MI) exhibit evidence of training (exaggerated LPS responses) and increased glycolysis, associated with increased HIF-1α gene expression and altered histone modification profile [77].

Whilst it is difficult to discern the precise impact of hypoxia signalling to training in human diseases, the presented literature supports its broad relevance. As with the preclinical data presented earlier, it is important to consider the context of hypoxia signalling, and it is likely that some hypoxic training states will also encompass factors such as metabolite depletion, lactate accumulation and inflammatory signalling (e.g. tissue infarction or abscess cavity), which could modify the outcome. Hence, a one size fits all approach to therapeutic modulation seems unlikely, and it may be that both beneficial and harmful outcomes could arise from the same stimulus (e.g. improved infection outcomes with augmented cancer progression) [74,76]. However, in regard to infection outcomes, exciting preclinical data indicate that prior exposure to hypoxia improves outcomes in mice with *S. aureus* infection and exposed to repeat hypoxia [66]. This was associated with altered myeloid metabolism suggestive of suppressed HIF signalling (in normoxia). Notably, myeloid cell HIF-1α knockout was able to mimic some of the protective effects of prior systemic hypoxia, and whilst it is unclear if this translated to improved survival, this work is the first to link hypoxia to immune training/tolerance in the context of infection. In the context of earlier data from myeloid HIF knockout mice, showing blunted pro-inflammatory cytokine responses to endotoxins, this may suggest a pathogenic role of excessive myeloid HIF signalling in sepsis [10,19,20]. Whether these insights are therapeutically tractable remains unclear, but agents targeting the HIF/PHD axis have already completed phase 3 clinical trials [78], and there is scope to apply these to alter innate immune behaviour [79,80]. Perhaps in the shorter term, understanding hypoxia signalling-induced functional and epigenetic responses in innate immune cells may allow biomarkers of altered immune responses in disease.

## Conclusions

**Figure 1.**
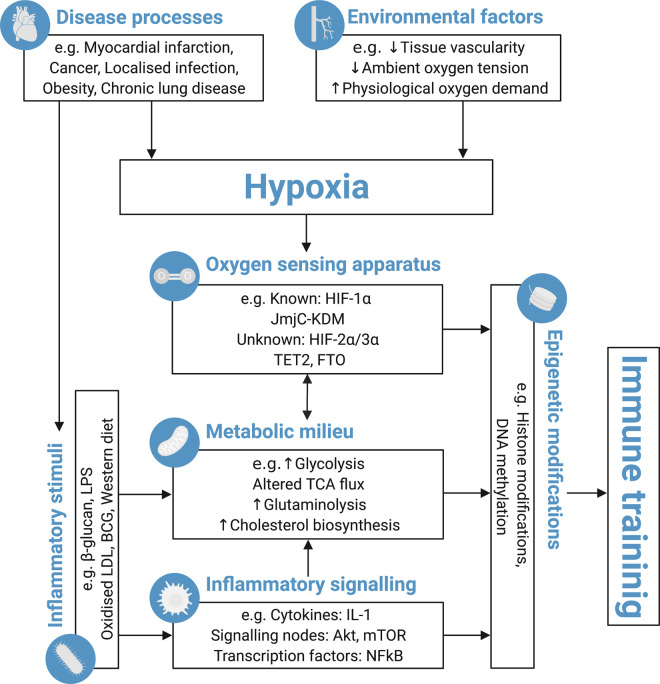
Schema demonstrating the proposed mechanisms by which the oxygen sensing apparatus of immune cells (and their stem/progenitor cells) modulates immune training. Disease processes and environmental factors integrate to modify cellular oxygen availability. However, the oxygen sensing apparatus are also influenced by cellular metabolic milieu, allowing activation of hypoxia sensing systems even under normoxic conditions. Epigenetic modifications represent an important integrator of these signals, which result in long-standing changes to innate immune responses i.e. ‘immune training’. Akt, Protein kinase B; BCG, Bacillus Calmette-Guerin; DNA, Deoxyribonucleic acid; FTO, Alpha-Ketoglutarate Dependent Dioxygenase; HIF, Hypoxia inducible factor; JmjC-KDMs, Jumonji C domain histone lysine demethylases; LDL, Low density lipoprotein; LPS, Lipopolysaccharide; mTOR, Mammalian target of rapamycin; NFkB, Nuclear factor kappa B; TCA, Tricarboxylic acid cycle; TET, Ten-eleven translocation. This figure was created with BioRender.com.

Hypoxia signalling contributes to long-term modification of innate immune responses, both arising from, and contributing to, a diverse range of disease processes. However, many questions remain about the underlying molecular mediators and how wider factors, such as metabolic state or inflammatory signalling milieu, integrate with hypoxia sensing (Figure 1). Greater understanding of these factors may allow us to move towards personalised therapeutic approaches to harness the beneficial elements of innate immune training.

## Perspectives

- The long-term behaviour of innate immune cells is influenced by prior exposure to diverse stimuli, that often induce metabolic rewiring and epigenetic modifications, in a process called ‘immune training’.
- Cellular hypoxia sensing pathways frequently regulate immune training, both during normoxic and hypoxic conditions. However, the role of more recently discovered oxygen sensing systems remains to be explored, as does the impact of hypoxic disease states upon the training process.
- Modifying immune training holds therapeutic potential for infectious and non-communicable diseases. Understanding how hypoxia signalling modulates training responses may offer an important avenue to achieve this therapeutic goal.

## Abbreviations

Akt: Protein kinase B
BCG: Bacillus Calmette Guerin
cAMP: Cyclic adenosine monophosphate
DNA: Deoxyribonucleic acid
ERK: Extracellular signal-regulated kinase
FIH: Factor inhibiting HIF
FTO: FTO alpha-ketoglutarate dependent dioxygenase
HIF: Hypoxia inducible factor
HSC: Haematopoietic stem cell
IFNγ: Interferon gamma
IKKβ: Inhibitor of nuclear factor kappa B kinase subunit beta
IL-1: Interleukin-1
iNOS: Inducible nitric oxide synthase
JmjC-KDMs: Jumonji C domain histone lysine demethylases
LDL: Low-density lipoprotein
LPS: Lipopolysaccharide
MI: Myocardial infarction
MPC: Myeloid progenitor cell
mTOR: Mammalian target of Rictor
NFkB: Nuclear factor kappa B
PHD: Prolyl hydroxylase domain
TET: Ten-eleven translocation
